# Divergent Response Strategies of CsABF Facing Abiotic Stress in Tea Plant: Perspectives From Drought-Tolerance Studies

**DOI:** 10.3389/fpls.2021.763843

**Published:** 2021-11-17

**Authors:** Jing Lu, Jinke Du, Liying Tian, Mengshuang Li, Xianchen Zhang, Shihua Zhang, Xiaochun Wan, Qi Chen

**Affiliations:** ^1^State Key Laboratory of Tea Plant Biology and Utilization, Anhui Agricultural University, Hefei, China; ^2^College of Life Science and Health, University of Science and Technology, Wuhan, China

**Keywords:** *Camellia sinensis* (L.), transcriptional regulation, cultivar variation, CsABFs, stress

## Abstract

In plants, the bZIP family plays vital roles in various biological processes, including seed maturation, flower development, light signal transduction, pathogen defense, and various stress responses. Tea, as a popular beverage, is widely cultivated and has withstood a degree of environmental adversity. Currently, knowledge of the bZIP gene family in tea plants remains very limited. In this study, a total of 76 *CsbZIP* genes in tea plant were identified for the whole genome. Phylogenetic analysis with *Arabidopsis* counterparts revealed that CsbZIP proteins clustered into 13 subgroups, among which 13 ABFs related to the ABA signaling transduction pathway were further identified by conserved motif alignment and named CsABF1-13, these belonged to the A and S subgroups of CsbZIP and had close evolutionary relationships, possessing uniform or similar motif compositions. Transcriptome analysis revealed the expression profiles of *CsABF* genes in different tissues (bud, young leaf, mature leaf, old leaf, stem, root, flower, and fruit) and under diverse environmental stresses (drought, salt, chilling, and MeJA). Several *CsABF* genes with relatively low tissue expression, including *CsABF1*, *CsABF5*, *CsABF9*, and *CsABF10*, showed strong expression induction in stress response. Thirteen *CsABF* genes, were examined by qRT-PCR in two tea plant cultivars, drought-tolerant “Taicha 12” and drought-sensitive “Fuyun 6”, under exogenous ABA and drought stress. Furthermore, *CsABF2*, *CsABF8*, and *CsABF11*, were screened out as key transcription factors regulating drought tolerance of tea cultivars. Subsequently, some potential target genes regulated by CsABFs were screened by co-expression network and enrichment analysis. This study update CsbZIP gene family and provides a global survey of the ABF gene family in tea plant. The resolution of the molecular mechanism of drought resistance in different varieties could be helpful for improving stress resistance in tea plant via genetic engineering.

## Introduction

Tea plants [*Camellia sinensis* (L.) O. Kuntze] originated in the southwest region of China and have been cultivated and utilized for thousands of years. Today, tea is widely grown worldwide (Reygaert, 2014). The tea plant is sensitive to environmental conditions and various adverse environmental factors, such as extreme temperature, drought, and salinity, can affect the growth of tea in the wild (Wang W. et al., 2016). Due to global climate change, the frequency of extreme weather such as high temperature and drought, has increased in recent years, causing serious harm to tea production. The study of the environmental tolerance of tea plants is helpful for improving the yield and quality of tea leaves. The potential stress resistance of plants is usually determined by the expression of stress-induced genes regulated by specific transcription factors (TFs) (Yamaguchi-Shinozaki and Shinozaki, 2006). TFs are widespread in plants and regulate the expression of target genes by binding directly or indirectly with specific *cis*-regulatory elements.

The basic (region) leucine zippers (bZIPs) are a class of TFs that are widely present in the life cycle of plants and play an important regulatory role, participating in many important biological processes, such as seed maturation, flower development, light signaling, pathogen defense, and response to various biotic and abiotic stresses (Wang et al., 2015). With respect to the number of TFs in the whole genome, the size of the bZIP-family varies considerably among species, with 78 in *Arabidopsis thaliana* (Droege-Laser et al., 2018), 91 in *Oryza sativa* (Liu et al., 2018), 247 in *Brassica napus* (Zhou et al., 2017), and 191 in *Triticum aestivum* (Agarwal et al., 2019).

Abscisic acid responsive element-binding factors (ABFs) are the most prominent subgroup in the A-bZIP family and have been found to act at the core of ABA signaling, which implements adaptive responses to counteract water deficits in vegetative tissues in response to abiotic stress, such as drought, salinity or extreme temperature (Droege-Laser et al., 2018). ABFs are regarded as a transit station for transmitting ABA signals to downstream functional genes. In short, members of the clade A serine/threonine protein phosphatases 2Cs (PP2Cs) have been found to negatively control ABA signaling by inactivating the SnRK2 (SNF1-related kinases 2) via dephosphorylation. ABA is captured by PYR/PYL/RCAR (pyrabactin resistance proteins/PYR-like proteins/regulatory component of the ABA receptor) co-receptors and forms ternary complexes with PP2C, thereby preventing their interaction with SnRK2 kinases (Fujii et al., 2009; Umezawa et al., 2010). SnRK2s directly phosphorylate five conserved serine/threonine kinase phosphorylation sites (RXXS/T) in ABFs to strongly enhance their transactivation properties (Takashi et al., 2006). Furthermore, ABFs regulate the expression of the ABA signaling pathway and other downstream stress-responsive target genes via direct binding to ABA responsive element (ABRE: ACGTGG/TC) *cis*-elements (Choi et al., 2000).

In recent years, several ABFs involved in plant response to drought stress have been identified. MEABL5, a transcription factor belonging to the A-clade of the bZIP family in *Manihot esculenta Crantz*, was found to specifically bind to the ABRE *cis*-element in the promoter of the major cell wall invertase gene *MeWINV3 in vitro and in vivo* to positively regulate drought tolerance (Liu et al., 2019). In *Brassica oleracea* (Zhou et al., 2013), BolABI5 was induced by ABA or drought stress and combined with ABRE *cis*-acting element in the promoter to resist drought stress. Overexpression of *AtABF3* in *Medicago sativa* can reduce the transpiration rate and accumulation of reactive oxygen species (ROS) and improve drought tolerance (Wang Z. et al., 2016). In *Ipomoea batatas*, overexpression of *IbABF4* can promote the root elongation of *Arabidopsis* seedlings under drought stress (Wang et al., 2019). *FtbZIP5* found in *Fagopyrum tataricum* can enhance the drought and salt resistance in transgenic *Arabidopsis*, mainly by regulating the accumulation of ROS and inducing the plant antioxidant system. In addition, *FtbZIP83* can respond to drought stress by up-regulating the transcription abundance of downstream ABA-induced genes, and overexpression of transgenic *Arabidopsis* was found to improve drought tolerance by reducing oxidative damage (Li et al., 2019; Li Q. et al., 2020).

We used the genomic data to identify the 76 *bZIPs* and then analyzed the expression level of these genes using RNAseq publicly available, 15 more than those reported by Hou et al. (2018). A phylogenetic tree was constructed with the *Arabidopsis bZIP* gene family as a reference. Then, based on the analysis of the evolutionary relationship and conserved motifs, 13 *ABF* genes related to the ABA signal transduction pathway were screened for transcriptome analysis. The expression patterns of *CsABFs* in eight different tea tissues and organs under different environmental stresses (drought, salt, chilling, and MeJA) were analyzed by transcriptome data analysis. The expression differences of *CsABFs* between drought-sensitive “Fuyun 6” and drought-tolerant “Taicha 12” were further compared in order to screen key TFs regulating the drought resistance of *C. sinensis* and to identify potential regulatory candidate genes using co-expression network analysis. This study provides a new concept of the structure and function of the ABF gene in tea plants, valuable resources for improving the stress resistance of tea plants, and a basis for improving the quality of tea.

## Materials and Methods

### Plant Materials and Abiotic Stress Treatment

Two-year-old tea cutting seedlings (Fuyun 6# and Taicha 12#) were obtained from Qianhe Tea Seedling Co., Ltd. in Quanzhou, Fujian Province. Seedlings with a height of 30–35 cm, complete root systems, and consistent growth were screened for domestication culture. Transition culture was performed with clean water and 25% Shigeki Konish nutrient solution every 7 days. Finally, the seedlings were transferred to the phytotron for hydroponic culture, and the conditions were as follows: illumination duration of 16 h light and 8 h dark alternately, room temperature of 23°C ± 1, air humidity of 45–50%, ventilation at intervals of 12 h, and the nutrient solution was replaced every 3 days.

After the tea cutting seedlings grew new fibrous roots, 0.25 mM ABA was added to the culture solution. The second and third leaves were sampled after treatment for 0, 0.5, 6, and 24 h and then immediately placed in liquid nitrogen. In addition, 25% PEG-6000 was added to the culture solution to perform the drought treatment, the second and third leaves were sampled after treatment for 0, 1, and 3 days. The control group was cultured in normal nutrient solution. Total samples were stored at −80°C for qRT-PCR detection. Three biological replicates of each sample were taken for subsequent testing.

### Chlorophyll Fluorescence Detection

The leaves of Fuyun 6# and Taicha 12# treated with drought for 0, 1, 3, and 5 days were measured by a portable pulse-modulated chlorophyll fluorescence analyzer (PAM 2500, Zealquest Scientific Technology Co., Ltd., Shanghai, China). Five sample leaves were tested in each treatment, and the results were averaged. First, the leaf was clamped by a dark adapted leaf clip. After 30 min of dark adaptation, the leaf was measured with IMA-min/B and blue at 450 nm. Then the initial fluorescence (F_o_), maximum fluorescence (F_m_), variable fluorescence (F_v_), and photosystem II (PSII) maximum light energy conversion rate (F_v_/F_m_) were obtained. The calculation formula used was as follows:


F/vF=m(F-mF)o/Fm

### Total RNA Extraction and qRT-PCR Analysis

Total RNA was extracted by the RNA-prep pure plant kit (polysaccharide and polyphenolic rich, TIANGEN, Shanghai, China), and reverse transcription was performed using the Goldenstar^TM^ RT6 cDNA Synthesis Kit Ver.2 (TSINGKE, Beijing, China). The *CsGAPDH* gene was applied as an internal reference for real-time quantitative PCR (qRT-PCR) analysis. Suitable primers were designed for all selected genes by Primer plus 3.0^1^, and only primers with a single melting curve and the amplification efficiency between 95–105% were selected for subsequent qRT-PCR analysis. All screened gene-specific primers are listed in Supplementary Table 2. The 2 × TsingKe Master qPCRmixKit (TsingKe, Beijing, China) was used for qRT-PCR on the QuanStudio 6-Flex fluorescence quantitative PCR platform (Thermo Fisher Scientific, Singapore). Every sample was tested in three biological replicates and three technical replicates, which showed good consistency, and the relative expression level was calculated using the 2^–ΔΔ^ *^*C*^*^*T*^ method.

### Identification and Biological Analysis of CsbZIP and CsABF

To screen the *bZIP* genes in the *Camellia sinensis* genome, the reported *AtbZIP* sequences were downloaded from the *Arabidopsis* gene annotation database TAIR^2^. Based on 78 AtbZIP protein sequences, a blast search was performed in the *C. sinensis* genome annotation database of TPIA^3^ (Xia et al., 2019) to obtain homologous CsbZIP coding sequences, genomic sequences and peptide sequences. Seventy-six annotated bZIP protein sequences were obtained. The integrity of the bZIP domain of each protein sequence was verified by SMART^4^ and PFAM^5^ and confirmed as a member of the *bZIP* gene family. Through the KEGG pathway on the TPIA website, 13 ABF transcription factors related to ABA signal transduction pathway were identified and named CsABF1–13 (Supplementary Table 1).

To investigate the phylogenetic relationship between the CsABF and CsbZIP gene families in tea plant, 78 bZIP in *Arabidopsis* were applied to construct the phylogenetic tree with 76 bZIP in *C. sinensis*. The phylogenetic tree was generated and displayed by the Mega 7.0 program with the following parameters: neighbor-joining (NJ) method, P-distance model, pairwise deletion, and 1000 bootstrap replicates. Multiple sequence alignments of CsABF were performed by DNAMAN. The Multiple Em for Motif Elicitation (MEME) suite (version 4.10.1)^6^ was used to identify the conserved motifs in all CsABFs. The parameters were as follows: maximum number of motifs: 6; number of repeats: any; maximum number of motifs: 50; optimal motif width: 6–200 amino acid residues.

### Transcriptome Data Processing and Expression Pattern Analysis

The transcriptome data of the tissue-specific expression of *CsABFs* were downloaded from the TPIA website, and the eight organs included apical bud, young leaf, mature leaf, old leaf, stem, root, flower, and fruit in appropriate seasons. The transcriptome data of CsABFs in response to drought and salinity stress (Zhang et al., 2017), chilling stress (Wang et al., 2013), and MeJA treatment (Shi et al., 2017) were obtained from NCBI. The data acquisition and homogenization processing methods refer to Li M. et al. (2020) (Supplementary Table 3). Multiple Experiment Viewer 4.9 software was used for heatmap clustering analysis.

### Construction of Co-expression Network and Prediction of Functional Genes

Co-expression analysis was applied in the tea plant drought stress transcriptome to construct the network related to *CsABFs*, which were then intersected with the genes screened from TeaCon website^7^ to narrow down the target range. Co-expressed genes were screened with the corresponding Pearson correlation coefficients (PCC-value) of more than 0.7 and then were used to construct the co-expressed network of *CsABFs* with Cytoscape 3.6.1 (Shannon et al., 2003). Some genes co-expressed by key transcription factors were selected for functional enrichment analysis in order to identify potential regulatory target genes (Supplementary Table 4). GO enrichment analysis was carried out by R language 3.6.3 (Bell Laboratories).

### Data Source and Statistical Analysis

One-way analysis of variance (ANOVA) was performed on the experimental data using SPSS 22 (IBM, United States), and multiple comparisons were performed using Duncan test to analyze the significance of the difference between the two treatments at the 0.05 level.

## Results and Analysis

### Identification and Phylogenetic Analysis of CsbZIP Family Members in Tea Plant

According to the reported *AtbZIP* genes as queries, 76 *bZIP* genes were identified from the latest assembled version database of the tea plant information archive (TPIA^8^). The integrity of all the bZIP domains was determined using the online tool SMART (see Text Footnote 4). The relevant information is listed in Supplementary Table 1.

**FIGURE 1:**
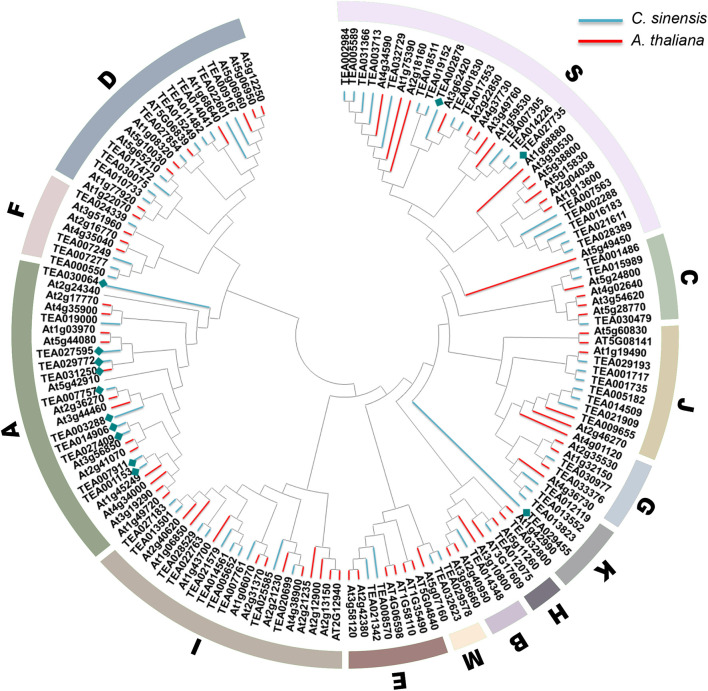
Phylogenetic tree. Consensus tree of bZIP proteins from *Camellia sinensis* and *Arabidopsis thaliana*. The conserved domain sequences were aligned by DNAMAN, and the phylogenetic tree was constructed using MEGA 7.0 by the neighbor-joining (NJ) method with 1,000 bootstrap replicates. Molecular analyses divided the bZIP into thirteen distinct clades. Clades A–K, M, and S are indicated by the colored areas. Blue lines represent CsbZIP, pink lines represent AtbZIP, and blue diamond represent the CsABF genes family member.

The bZIP transcription factor gene family is huge and complex, but all members contain the typical leucine zipper region N-X7-R/K-X9-L-X6-L-X6-L (Jakoby et al., 2002). Droege-Laser et al. (2018) divided 78 members of *Arabidopsis bZIP* gene family into the 13 subfamilies of A, B, C, D, E, F, G, H, I, J, K, M, and S according to the basic domain and other conserved domains. Subgroup A is mainly involved in ABA and stress signal transduction, subgroup C is mainly involved in seed development and pathogen defense, subgroup D is involved in disease defense and physiological growth, and subgroup H plays a key role in photosynthesis; subfamily S is the largest bZIP subfamily in *Arabidopsis* and is involved in sugar signaling and stress pathways (Jakoby et al., 2002). To elucidate the phylogenetic relationships of the bZIP family in tea plant, an unrooted tree was established based on the aligned 76 CsbZIPs with 78 AtbZIP protein sequences (Figure 1). The results indicated that most of the obtained subgroups were consistent with previous phylogenetic analyses (Hou et al., 2018). As shown in Figure 1, the phylogenetic tree clustered all of the CsbZIP members into 13 subgroups similarly to AtbZIPs. In addition, thirteen ABF genes related to ABA signal transduction were identified and named CsABF1–13. Noteworthy, CsABF1–13 were mainly clustered in the A subgroup (specifically CsABF1/2/3/5/6/7/8/11/12/13), meanwhile CsABF10 and CsABF4/9 clustered in subgroup K and S, respectively (blue diamond). Most of the genes in the A and S subgroups of the ABA-activated signaling pathway are ABF (ABRE binding factor) or AREB (ABA response element binding protein) proteins, which can bind to different ABRE-containing promoters (Choi et al., 2000; Uno et al., 2000). Under all stress conditions, the differentially expressed genes were enriched in the A and S subgroups, indicating that these two subgroup members might act as key factors in stress responses (Wang et al., 2020).

### Gene Structure and Expression Profile of the CsABF Gene Family

The function of ABA in plants depends on signal perception and transduction. Existing research has shown that when plants suffer stress due to adversity, especially drought stress, the body increases ABA synthesis levels, senses, and transmits signals through the PYL-PP2C-SnRK2 complex. ABF transcription factors are activated to regulate the expression of downstream genes, thus enabling plants to adapt to harsh environments (Fahad et al., 2017). In order to better explore the response strategies of tea plant to drought stress, we focused on the ABFs, which are closely related to ABA signal transduction in the bZIP family. The diversity of plant protein sequences and structures generated in biological evolution is the possible mechanism for the formation of polygene families and functional diversity. The characteristics of plant diversity lead to the efficient utilization of natural resources or adaptation to adverse environments (Shang et al., 2013). Therefore, we performed multiple sequence alignment for 13 full-length CsABF protein sequences using DNAMAN and predicted the conserved motifs and their phylogenetic relationships with MEME in order to further understand the structural features of the ABF proteins in tea plant.

**FIGURE 2:**
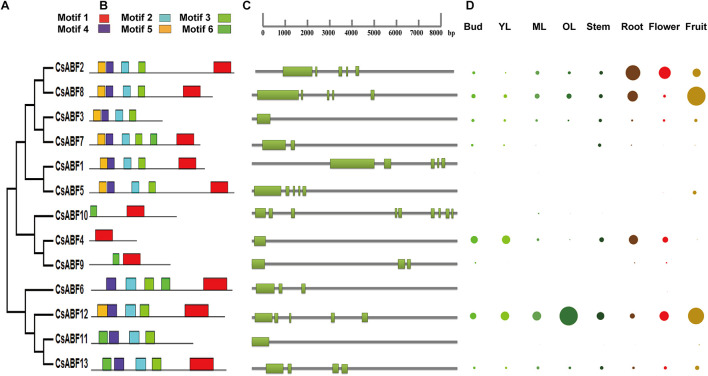
Characteristics of the *CsABF* gene family members. **(A)** Phylogenetic tree. **(B)** Conserved motifs in the CsABFs. **(C)** Genetic structure analysis. **(D)** Tissue-specific expression. Different colors represent different organs, and the circle size represents the relative expression level. YL, young leaves (1st and 2nd leaves); ML, mature leaves (3rd and 4th leaves); OL, old leaves. The relative expression data were log2 transformed and diagram was drawn using the Multiple Array Viewer software.

Phylogenetic analyses of CsABFs were performed (Figure 2A), and corresponding genetic structure analyses by the GSDS website were used to reveal the exon and intron structures of *CsABFs* (Figure 2C). It’s worth noting that the *CsABF3*, *CsABF4*, and *CsABF11* genes did not have any introns in their genomic sequences. Other *CsABF* genes contain anywhere from one to multiple introns each. These differences indicate that *CsABFs* are complex in evolution, and have different expression regulation modes.

Six conserved motifs were identified in CsABFs with widths ranging from 6 to 50, and the locus distribution of each sequence was zero or one. The height of each letter represents the conservatism of a specific amino acid in each motif (Figure 2B). Similar to other plants, Motif1 is typical in bZIP domains detected by PFAM. The basic domain is close to the N-terminal of the leucine zipper domain and acts as a nuclear localization signal by binding the fixed N-X7-R/K structure with a specific DNA sequence (Landschulz et al., 1988). Most of the CsABF protein sequences share common motifs, but the conserved domains of *CsABF4*, *CsABF9*, and *CsABF10* are obviously different from others (Figure 2B), which may be the reason why they do not belong to the A subfamily of CsbZIP, which also means that they have genetic functional diversity. This result can provide a reference for further studies on functional differentiation among bZIP subfamilies.

Based on TPIA transcriptome data, the differential expression profile of *CsABF* genes in eight tissues was analyzed. The relative expression of all genes in a single tissue is shown in Figure 2D. The expression levels of *CsABF12* were higher in old leaf, those of *CsABF2*, *CsABF4*, and *CsABF8* were highly expressed in root, and *CsABF2*, *CsABF8*, and *CsABF12* were highly expressed in fruit, which suggests that the *CsABFs* have functional diversification. There are also some gene family members with very low expression that may be regulated by different developmental processes and stresses. When plants suffer from drought stress, the root is the first to feel a water deficit, and the root stem cell niche, meristem and vascular system will coordinate their response to drought. Taking into account that *CsABF2* and *CsABF8* are both expressed in roots and fruits, it may be worthwhile to investigate their roles in the regulation of drought resistance in tea plants.

### Expression Pattern of CsABFs Under Different Abiotic Stress

**FIGURE 3:**
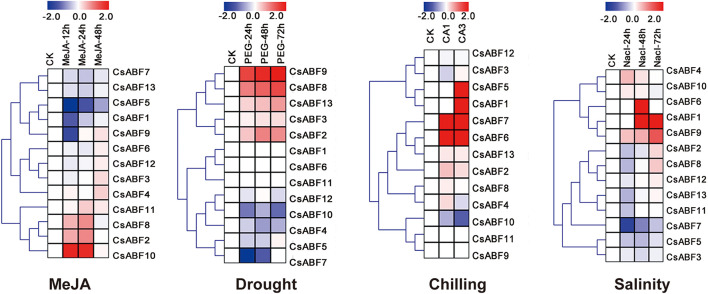
Heatmap of CsABF gene expression under different abiotic stress treatments. Stress treatments include methyl jasmonate (MeJA), drought (PEG), chilling and salinity stress. The FPKM values of the Illumina RNA-seq data were reanalyzed and log2 transformed. The color scale represents the gene expression level; blue represents down-regulation, and red represents up-regulation.

In order to analyze the different response strategies of *CsABFs* to different abiotic stresses, transcriptome data of tea plant under different stress treatments were downloaded from NCBI, including drought, salinity, chilling, and MeJA treatment. After homogenizing the RPKM values of corresponding unigenes, a heatmap was drawn for further analysis. From Figure 3, it can be seen that divergent response strategies of *CsABFs* to face abiotic stresses. The expression levels of *CsABF2*, *CsABF8*, and *CsABF10* were significantly up-regulated from 12 to 24 h after MeJA treatment and then decreased to normal levels. *CsABF1*, *CsABF5*, and *CsABF9* were significantly down-regulated within 12 h after treatment, and the down-regulation of *CsABF5* expression persisted for a long time, indicating the negative regulation of MeJA signaling. Under drought treatment, the expression levels of *CsABF2*, *CsABF8*, *CsABF9*, and *CsABF13* were significantly up-regulated, while the expression levels of *CsABF4*, *CsABF7*, and CsABF10 *w*ere decreased. Under cold stress treatment, the expression levels of *CsABF6* and *CsABF7* were significantly up-regulated during full acclimation (CA1) and de-acclimation (CA3), while *CsABF1* and *CsABF5* showed no significant changes in CA1, but were increased in CA3. The expression of *CsABF10* showed significant down-regulation in both CA1 and CA3. Under salt treatment, the expression levels of *CsABF1*, *CsABF6* and *CsABF9* were increased, and *CsABF6* reached the highest expression level at 48 h. The expression level of *CsABF7* obviously decreased. The results showed that there was no redundancy in the stress responses at transcriptional level of the *CsABF* family members, and no single gene could deal with more than two stresses. Based on our analysis, we consider that each gene show a specific answer to the stresses imposed and they might regulate downstream genes to deal with a complex environment.

### Effect of Exogenous ABA and Drought Stress on CsABFs Expression in Different Tea Cultivars

**FIGURE 4:**
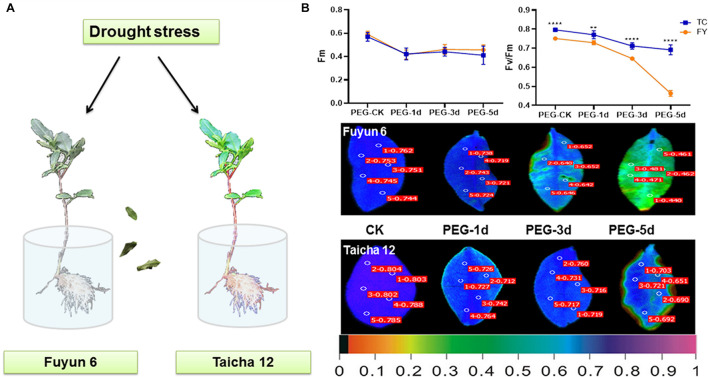
**(A)** Schematic diagram of different tea varieties to drought. **(B)** Chloroplast fluorescence parameters of *F*_*m*_, *F*_*v*_/*F*_*m*_, and other leaves of different tea varieties under drought treatment. The false color code depicted at the bottom of the image ranged from 0 (black) to 1.0 (purple). *t*-test analysis, **p < 0.005, ****p < 0.0001.

According to earlier study, the drought-sensitive variety “Fuyun 6” and drought-tolerant variety “Taicha 12” were screened from many tea varieties (Zhang et al., 2019). In this experiment, they were selected for further studies on the changes of ABF gene family expression patterns during drought and ABA stress treatments. By phenotypic observation and chlorophyll fluorescence monitoring, it can be seen that after 3 days of drought treatment, the leaves of “Fu Yun 6” showed an obvious dehydration phenotype and gradually dried up, while the dehydration of leaves of “Taicha 12” was only observed on the fifth day of drought stress (Figure 4A). *F*_*m*_ represents the maximum fluorescence value that can be absorbed by plants (Dong et al., 2020), and its change is affected by environment (Lu and Lu, 2020). In addition, *F*_*v*_/*F*_*m*_ is also an important index to study the effects of various environmental stresses on photosynthesis (Li et al., 2014). As shown in Figure 4B, Fm of both tea varieties showed the same slight decreasing trend under drought stress. However, from the third day of drought treatment, *F*_*v*_/*F*_*m*_ dropped sharply in “Fuyun 6” but had no significant change in “Taicha 12” indicating that “Taicha 12” had significantly higher drought resistance than “Fuyun 6”. This difference was also evident in the leaf fluorescence diagram. The results indicate that the drought resistance of the two tea varieties does exist, providing powerful test materials for the subsequent investigation of molecular mechanisms of drought regulation.

**FIGURE 5:**
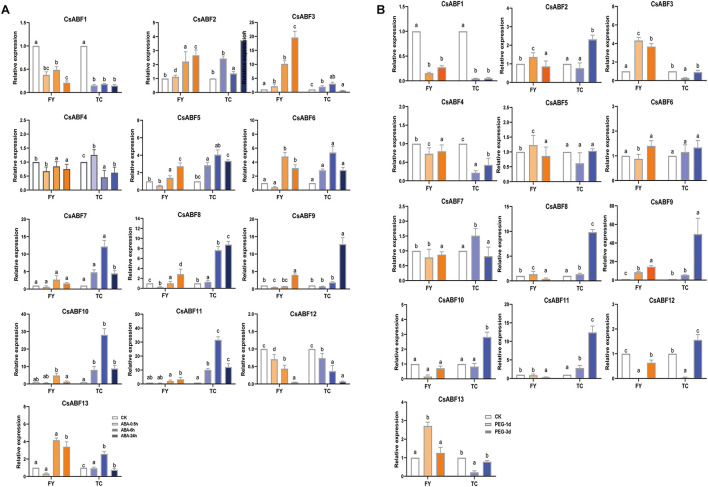
qRT-PCR analysis the change of relative expression of *CsABF* genes. **(A)** Expression profiles of *CsABFs* in tea plant cultivars “Fuyun 6” (FY) and “Taicha 12” (TC) under exogenous ABA treatment. **(B)** Expression profiles of *CsABFs* in tea cultivars “Fuyun 6” (FY) and “Taicha 12” (TC) under drought stress. Different letters (a, b, and c) indicate statistical significance among treatments using one-way analysis of variance (ANOVA) test and a Fisher’s least significant difference (LSD) at the 5% significance level.

In order to analyze the molecular mechanism of different tea varieties in response to drought stress, and to explore the key factors involved in the regulation of the *CsABF* genes family, drought-sensitive variety “Fuyun 6” and drought-tolerant variety “Taicha 12” were selected as experimental materials to undergo exogenous ABA and drought stress treatment. The expression patterns of *CsABFs* in different tea cultivars were detected by qRT-PCR. As can be seen from Figure 5A, all family members except *CsABF1*, *CsABF4*, and *CsABF12* showed a significant up-regulation trend after ABA treatment. The difference between the two tea cultivars is that *CsABF7*, *CsABF8*, *CsABF9*, *CsABF10*, and *CsABF11* induced significantly higher up-regulated expression in “Taicha 12” than in “Fuyun 6”, while *CsABF3* showed a much higher expression level in “Fuyun 6” than in “Taicha 12”. These results suggested that some *CsABF* gene family members exhibited completely different expression patterns in two different tea cultivars, implying that they may be potentially key resistance factors by regulating downstream genes and generating different resistance responses. The response model of CsABF family members to drought stress was very similar to the results after exogenous ABA treatment, and the expression changes of some genes showed a certain lag effect considering that the signaling conduction induced by exogenous ABA was significantly faster than the endogenous signaling conduction induced by drought stress.

As shown in Figure 5B, the expression levels of *CsABF2*, *CsABF8*, *CsABF9*, *CsABF10*, and *CsABF11* reached the highest point within 6–24 h in “Taicha 12” after exogenous ABA treatment and then decreased. They did not change significantly at first in drought-treated “Taicha 12” until their expression levels reached the highest point 72 h after treatment, indicating that they both responded to ABA signal transduction caused by drought. However, they changed less in “Fuyun 6”. It is thus speculated that when “Taicha 12” is subjected to drought stress, the expression of these genes will be activated to initiate downstream functional genes to exercise power and then promote the enhancement of drought tolerance in tea plant, which is the key for “Taicha 12” to become a drought-tolerant variety. Therefore, the key transcription factors from these genes for tea plants to cope with drought were screened for follow-up studies.

### Co-expression Network

The gene co-expression network can be divided into a condition-dependent network and a condition-independent network according to the experimental treatment (Proost and Mutwil, 2016). Condition-dependent gene networks are useful for revealing the relationship between genes under a specific condition such as biotic or abiotic stress. Weighted gene co-expression networks have proven to be a valuable tool for revealing unknown gene relationships in condition-dependent models (Zhang et al., 2020).

**FIGURE 6:**
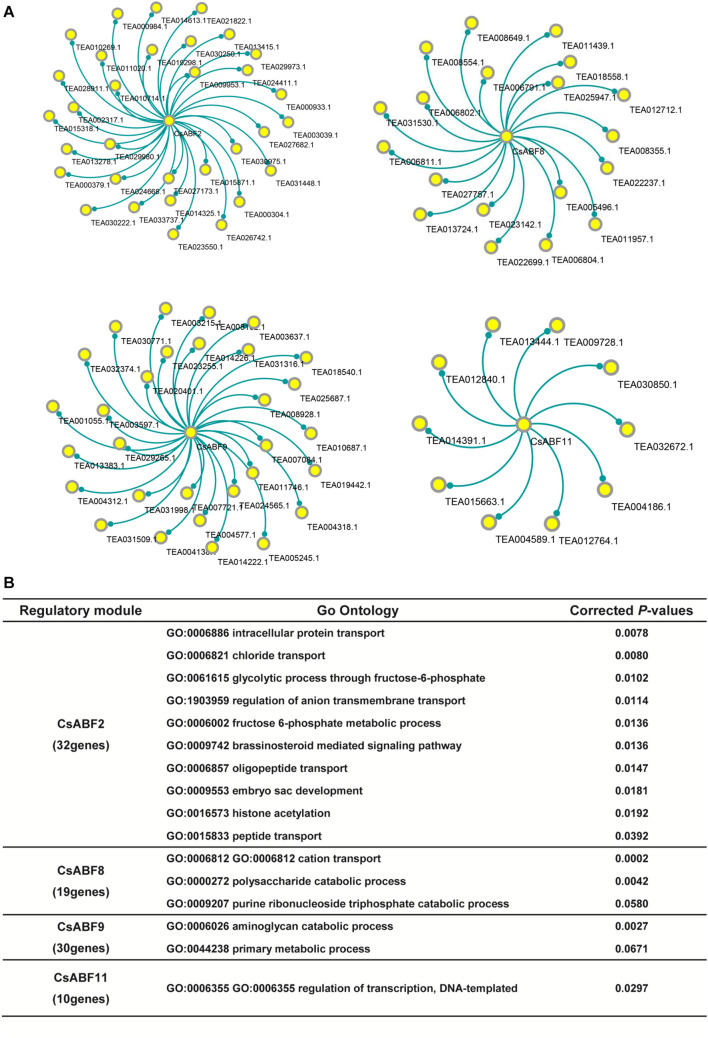
**(A)** Co-expression network analysis of key TFs. The core loci are *CsABF2*, *CsABF8*, *CsABF9*, and *CsABF11*. **(B)** GO enrichment analysis table of candidate genes regulated by TF CsABF2/8/9/11. GO analysis will return a *P*-value for each GO with a differential gene, and a small *P*-value means that the differential gene is enriched in the GO.

The co-expression genes of *CsABF2*, *CsABF8*, *CsABF9*, and *CsABF11* were screened out and network visualization was constructed as shown in Figure 6A. According to the same PCC-value, we screened 32, 19, 30, and 10 functional genes that were highly correlated with *CsABF2*, *CsABF8*, *CsABF9*, and *CsABF11*, respectively. The results of the co-expression network showed that although these four ABF transcription factors should stimulate the drought tolerance of “Taicha12” varieties, they were independent of each other, and there were no functional genes that acted synergistically. This indicates that tea plants have a complex molecular regulation mechanism to cope with drought stress. Among these co-expression networks, some of the genes are potentially stress-responsive genes regulated by ABF transcription factors, which need to be characterized by further studies. As shown in Figure 6B, among these candidate genes, *CsABF2* may be involved in regulating transport and transduction processes, including intracellular protein transport, oligopeptide transport, and chloride transport, and may also be involved in brassinosteroid mediated signal regulation; *CsABF8* may be involved in the regulation of energy metabolism processes such as the cation transport and the polysaccharide catabolic process; *CsABF9* may be involved in the amino-glycan catabolic process; *CsABF11* may be involved in regulating DNA-templated transcription. Meanwhile, most of the downstream target genes screened were hypothetical proteins that had not yet been named or verified, which posed a challenge for the subsequent verification work.

## Discussion

With the development of bioinformatics technology, the genome sequencing and assembly results for tea plant have become increasingly complete. Transcriptome sequencing has great potential in the discovery and identification of new genes. The bZIP transcription factor family in tea plant has been identified, with 18 genes (Cao et al., 2015) identified at the beginning, increasing to 61 genes (Hou et al., 2018), and finally to 76 genes in the present study. This promoted our exploration of the regulatory functions of bZIP-TFs. As a perennial woody plant, tea has wide distribution and a rich variety of resources, and it has developed unique adaptive environmental regulation mechanisms during evolution (Shen et al., 2019). Among them, the molecular mechanism of CsABF regulating the drought resistance of tea plant is worthy of further exploration. In this study, a phylogenetic tree comparison of CsbZIP and its corresponding homologous *AtbZIP* sequences was performed to explore the *CsbZIP* genes family at the genome-wide level, and the subfamily of the CsABF TFs involved in the ABA signal transduction pathway was identified. The transcriptome data of the *CsABF* genes in diversified stress treatments were analyzed, as well as the qRT-PCR data of gene expression patterns in drought-sensitive “Fuyun 6” and drought-tolerant “Taicha 12” under drought stress. CsABF2, CsABF8, CsABF9, and CsABF11 were screened out as key TFs in response to drought stress, and their potential downstream regulatory target genes were screened out by the co-expression network.

### Co-expression Network Provides Powerful Support for Exploring the New Function Genes

Co-expression network analysis can reveal potential associations between genes and regulatory factors, which undoubtedly helps us to search for potential downstream regulatory genes of CsABF and uncover the association between them to co-create the response network of drought stress. All candidate genes in Figure 6 were further validated by qRT-PCR to confirm that their expression patterns were indeed consistent with the corresponding ABF TFs. Therefore, mining the functions of these genes will help us understand how woody plants differ from herbaceous plants in responding to drought stress.

Sequence alignment of the candidate genes screened by the co-expression network revealed that there were 32 highly correlated functional genes with *CsABF2*, among which TEA000304.1 belonged to the NRT1/PTR gene family, and the function of homologous gene *AtNPF4.6* as a hormone transporter was verified in *A. thaliana. AtNPF4.6* was initially identified as nitrate transporter NRT1.2, but it was also identified as an ABA transporter (Chiba et al., 2015). *AtNPF4.6* has been proven to transport ABA to guard cells, thereby closing stomata to reduce water loss caused by transpiration and improving the drought resistance of *Arabidopsis*. Three other members of the *AtNPF* gene family, *AtNPF4.1*, *AtNPF4.2*, and *AtNPF4.5*, have also been shown to transport ABA in yeast (Kanno et al., 2012). Therefore, it is proposed that TEA000304.1 is regulated by CsABF2 and plays a role in the process of drought stress resistance in the “Taicha 12” cultivar.

Among the highly relevant candidate genes by *CsABF8*, TEA008554.1 was found to have high homology with β-amylase I (BamI) in *Arabidopsis*. AtBamI can participate in the daily degradation of starch in guard cells to maintain stomatal openings. Under drought stress, AtBamI can also generate a carbon skeleton to support proline biosynthesis through the daily degradation of instantaneous starch, so as to cope with the osmotic pressure imbalance caused by drought and ensure the normal growth of plants. Therefore, we speculate that TEA008554.1 is involved in regulating the physiological process of starch degradation in tea leaves under drought treatment.

Plants under drought stress can produce osmoprotective agents, such as sucrose, proline, LEA protein and antioxidant enzymes (Szabados and Savouré, 2010). Under the protection of osmotic agents, protein denaturation, membrane damage and reactive oxygen damage were reduced in plants, so as to maintain normal cell turgor and improve the drought resistance of plants. This study revealed that many putative proteins were involved in the process of CsABF regulating drought resistance. It was speculated that CsABF2 and CsABF8 were mainly responsible for defense by regulating polysaccharide metabolism and transporter proteins, while CsABF11 was involved in stress response mainly through the regulation of the DNA transcription process. However, the function of these unknown genes remains to be demonstrated in follow-up research.

### Drought-Tolerant Cultivars Provide a Perspective for Studying Tea Plant Response Strategies to Abiotic Stress

The diversification of plant resources has allowed them to evolve different strategies in response to environmental stress. Many species have been screened for drought-sensitive and drought-tolerant varieties to study the differences in molecular regulation mechanisms. According to current research reports, screening is mainly based on plant phenotypes, physiological data, and changes in metabolite content *in vivo*, and there is no consistent molecular label that can be used to distinguish drought-sensitive and drought-tolerant varieties. In *T. aestivum*, the drought-tolerant variety “Luhan 7” can resist drought by responding to light-signaling pathways, while the drought-sensitive “Yangmai 16” variety can improve the yield through pre-drought induction to increase post-flowering drought stress tolerance during the vegetative period (Abid et al., 2016). In *Solanum lycopersicum*, drought resistance can be improved by reducing chlorophyll concentration and light absorption by chloroplasts (Sánchez-Rodríguez et al., 2012). In *Foeniculum vulgare* Mill, the abundance of glycol-degradation related proteins in the drought-sensitive type decreased in response to drought stress, while those in the drought-tolerant type increased in response to drought stress (Khodadadi et al., 2017).

**FIGURE 7:**
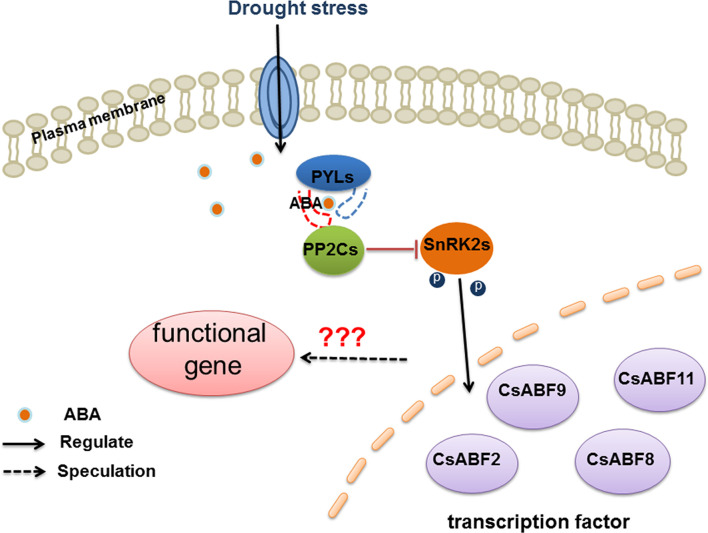
Model diagram of CsABFs responds to drought stress through the ABA signaling pathway.

The ABA signal transducing pathway is an important pathway of drought stress signal transmission. As shown in Figure 7, there may be regulatory factors highly responsive to ABA signal in different tea varieties, which are involved in regulating downstream functional genes to build stress resistance network to deal with the change of environment. Zhang et al. (2019) identified drought-sensitive and drought-tolerant varieties in tea plant. We conducted research on the molecular mechanism of ABA signaling in response to drought in tea pant in the early stage, but the drought-resistance mechanism of downstream transcription factors remains to be further studied. There are many reports on the involvement of different TFs in drought stress regulation. For example, in *T. aestivum*, TaCIPK27 partly plays a positive regulatory role to improve the drought resistance of wheat through the ABA-dependent pathway under drought stress (Wang et al., 2018). In *A. thaliana*, AtWRKY46 can independently regulate osmotic stress response and stomatal movement to resist drought stress. In *Gossypium hirsutum*, GbMYB5 could increase proline content, antioxidant enzyme activity and malondialdehyde (MDA) content to resist drought stress (Chen et al., 2015). In *O. sativa*, ONAC022 plays a positive role in drought stress tolerance through modulating an ABA-mediated pathway (Hong et al., 2016). OsNAC14 mediates drought tolerance by recruiting factors involved in DNA damage repair and defense responses, thereby improving drought tolerance in rice (Shim et al., 2018). In addition, the transcription factor OsNF-YA10 enhanced drought tolerance in rice (Yang et al., 2017). There are many reports on the involvement of different transcription factors in the regulation of drought stress, but whether transcription factors are the key factors for the formation of different drought-resistant varieties of tea remains to be further studied.

In this study, by constructing the potential regulatory network of CsABFs in different varieties (drought-sensitive and drought-tolerant), we gained a better understanding of the role of TFs in key varieties of crops and provided a theoretical basis for breeding drought-resistant varieties at the molecular level.

## Data Availability Statement

The datasets presented in this study can be found in online repositories. The names of the repository/repositories and accession number(s) can be found in the article/Supplementary Material.

## Author Contributions

JL conducted the experiments and drafted the manuscript. JD analyzed the transcriptome data. LT and ML conducted part of the qRT-PCR experiment. XZ provided the selected experimental materials. SZ directed the construction of transcription factor co-expression network. XW designed the experiments. QC directed the experiments and revised the manuscript. All authors have read and approved the final manuscript.

## Conflict of Interest

The authors declare that the research was conducted in the absence of any commercial or financial relationships that could be construed as a potential conflict of interest.

## Publisher’s Note

All claims expressed in this article are solely those of the authors and do not necessarily represent those of their affiliated organizations, or those of the publisher, the editors and the reviewers. Any product that may be evaluated in this article, or claim that may be made by its manufacturer, is not guaranteed or endorsed by the publisher.

## Footnotes

1
http://www.primer3plus.com/cgi-bin/dev/primer3plus.cgi

2
http://www.arabidopsis.org/

3
http://tpia.teaplant.org/

4
http://smart.embl-heidelberg.de/

5
http://pfam.xfam.org/

6
http://meme-suite.org/tools/meme

7
http://teacon.wchoda.com./

8
http://tpdb.shengxin.ren/

## References

[B1] Abid M. Tian Z. Ata-Ul-Karim S. T. Liu Y. Cui Y. Zahoor R. (2016). Improved tolerance to post-anthesis drought stress by pre-drought priming at vegetative stages in drought-tolerant and -sensitive wheat cultivars. *Plant Physiol. Biochem.* 106 218–227. 10.1016/j.plaphy.2016.05.003 27179928

[B2] Agarwal P. Baranwal V. K. Khurana P. (2019). Genome-wide analysis of bZIP transcription factors in wheat and functional characterization of a tabZIP under abiotic Stress. *Sci. Rep.* 9 4525–4608. 10.1038/s41598-019-40659-40657 30872683 PMC6418127

[B3] Cao H. Wang L. Yue C. Hao X. Wang X. Yang Y. (2015). Isolation and expression analysis of 18 CsbZIP genes implicated in abiotic stress responses in the tea plant (*Camellia sinensis*). *Plant Physiol. Biochem.* 97 432–442. 10.1016/j.plaphy.2015.10.030 26555901

[B4] Chen T. Li W. Hu X. Guo J. Liu A. Zhang B. (2015). A cotton MYB transcription factor, GbMYB5, is positively involved in plant adaptive response to drought stress. *Plant Cell Physiol.* 56 917–929. 10.1093/pcp/pcv019 25657343

[B5] Chiba Y. Shimizu T. Miyakawa S. Kanno Y. Koshiba T. Kamiya Y. (2015). Identification of Arabidopsis thaliana NRT1/PTR FAMILY (NPF) proteins capable of transporting plant hormones. *J. Plant Res.* 128 679–686. 10.1007/s10265-015-0710-712 25801271

[B6] Choi H. I. Hong J. H. Ha J. O. Kang J. Y. Kim S. Y. (2000). ABFs, a family of ABA-responsive element binding factors. *J. Biol. Chem.* 275 1723–1730. 10.1074/jbc.275.3.1723 10636868

[B7] Dong Z. Men Y. Liu Z. Li J. Ji J. (2020). Application of chlorophyll fluorescence imaging technique in analysis and detection of chilling injury of tomato seedlings. *Comput. Electron. Agr.* 168 105109–105117. 10.1016/j.compag.2019.105109

[B8] Droege-Laser W. Snoek B. L. Snel B. Weiste C. (2018). The *Arabidopsis* bZIP transcription factor family–an update. *Curr. Opin. Plant Biol.* 45 36–49. 10.1016/j.pbi.2018.05.001 29860175

[B9] Fahad S. Bajwa A. A. Nazir U. Anjum S. A. Farooq A. Zohaib A. (2017). Crop production under drought and heat stress: plant responses and management options. *Front. Plant Sci.* 8:1147. 10.3389/fpls.2017.01147 28706531 PMC5489704

[B10] Fujii H. Chinnusamy V. Rodrigues A. Rubio S. Antoni R. Park S. Y. (2009). In vitro reconstitution of an abscisic acid signalling pathway. *Nature* 462 660–664. 10.1038/nature08599 19924127 PMC2803041

[B11] Hong Y. Zhang H. Huang L. Li D. Song F. (2016). Overexpression of a stress-responsive NAC transcription factor gene ONAC022 improves drought and salt tolerance in rice. *Front. Plant Sci.* 7:4. 10.3389/fpls.2016.00004 26834774 PMC4722120

[B12] Hou Y. Wu A. He Y. Li F. Wei C. (2018). Genome-wide characterization of the basic leucine zipper transcription factors in *Camellia sinensis*. *Tree Genet. Genome* 14 27–38. 10.1007/s11295-018-1242-1244

[B13] Jakoby M. Weisshaar B. Dröge-Laser W. Vicente-Carbajosa J. Tiedemann J. Kroj T. (2002). bZIP transcription factors in *Arabidopsis*. *Trends Plant Sci.* 7 106–111. 10.1016/s1360-1385(01)02223-2223 11906833

[B14] Kanno Y. Hanada A. Chiba Y. Ichikawa T. Nakazawa M. Matsui M. (2012). Identification of an abscisic acid transporter by functional screening using the receptor complex as a sensor. *Proc. Natl. Acad. Sci. U S A.* 109 9653–9658. 10.1073/pnas.1203567109 22645333 PMC3386071

[B15] Khodadadi E. Fakheri B. A. Aharizad S. Emamjomeh A. Norouzi M. Komatsu S. (2017). Leaf proteomics of drought-sensitive and drought-tolerant genotypes of fennel. *BBA-Proteins Proteom.* 1865 1433–1444. 10.1016/j.bbapap.2017.08.012 28887228

[B16] Landschulz W. Johnson P. McKnight S. (1988). The leucine zipper: a hypothetical structure common to a new class of DNA binding proteins. *Science* 240 1759–1764. 10.1126/science.3289117 3289117

[B17] Li H. A. O. Liu S. S. Yi C. Y. Wang F. Zhou J. I. E. Xia X. J. (2014). Hydrogen peroxide mediates abscisic acid-induced HSP 70 accumulation and heat tolerance in grafted cucumber plants. *Plant Cell Environ.* 37 2768–2780. 10.1111/pce.12360 24773056

[B18] Li M. Lu J. Tao M. Li M. Yang H. Xia E. H. (2020). Genome-wide identification of seven polyamine oxidase genes in *Camellia sinensis* (L.) and their expression patterns under various abiotic stresses. *Front. Plant Sci.* 11:544933. 10.3389/fpls.2020.544933 33013966 PMC7500180

[B19] Li Q. Wu Q. Wang A. Lv B. Dong Q. Yao Y. (2019). Tartary buckwheat transcription factor FtbZIP83 improves the drought/salt tolerance of *Arabidopsis* via an ABA-mediated pathway. *Plant Physiol. Biochem.* 144 312–323. 10.1016/j.plaphy.2019.10.003 31606716

[B20] Li Q. Zhao H. Wang X. Kang J. Lv B. Dong Q. (2020). Tartary buckwheat transcription factor FtbZIP5, regulated by FtSnRK2.6, can improve salt/drought resistance in transgenic *Arabidopsis*. *Int. J. Mol. Sci.* 21 1123–1138. 10.3390/ijms21031123 32046219 PMC7037857

[B21] Liu C. Ou S. Mao B. Tang J. Wang W. Wang H. (2018). Early selection of bZIP73 facilitated adaptation of japonica rice to cold climates. *Nat. Commun.* 9 3302–3323. 10.1038/s41467-018-05753-w 30120236 PMC6098049

[B22] Liu J. Chen X. Wang S. Wang Y. Ouyang Y. Yao Y. (2019). MeABL5, an ABA insensitive 5-like basic leucine zipper transcription factor, positively regulates MeCWINV3 in Cassava (*Manihot esculenta* Crantz). *Front. Plant Sci.* 10:772. 10.3389/fpls.2019.00772 31316528 PMC6609874

[B23] Lu Y. Lu R. (2020). Enhancing chlorophyll fluorescence imaging under structured illumination with automatic vignetting correction for detection of chilling injury in cucumbers. *Comput. Electron. Agr.* 168 105145–105153. 10.1016/j.compag.2019.105145

[B24] Proost S. Mutwil M. (2016). Tools of the trade: studying molecular networks in plants. *Curr. Opin. Plant Biol.* 30 143–150. 10.1016/j.pbi.2016.02.010 26990519

[B25] Reygaert W. C. (2014). The antimicrobial possibilities of green tea. *Front. Microbiol.* 5:434. 10.3389/fmicb.2014.00434 25191312 PMC4138486

[B26] Sánchez-Rodríguez E. Rubio-Wilhelmi M. D. M. Blasco B. Leyva R. Romero L. Ruiz J. M. (2012). Antioxidant response resides in the shoot in reciprocal grafts of drought-tolerant and drought-sensitive cultivars in tomato under water stress. *Plant Sci.* 18 89–96. 10.1016/j.plantsci.2011.12.019 22525248

[B27] Shang H. Li W. Zou C. Yuan Y. (2013). Analyses of the NAC transcription factor gene family in *Gossypium raimondii* Ulbr.: chromosomal location, structure, phylogeny, and expression patterns. *J. Integr. Plant Biol.* 55 663–676. 10.1111/jipb.12085 23756542

[B28] Shannon P. Markiel A. Ozier O. Baliga N. S. Wang J. T. Ramage D. (2003). Cytoscape: a software environment for integrated models of biomolecular interaction networks. *Genome Res.* 13 2498–2504. 10.1101/gr.1239303 14597658 PMC403769

[B29] Shen W. Li H. Teng R. Wang Y. Wang W. Zhuang J. (2019). Genomic and transcriptomic analyses of HD-Zip family transcription factors and their responses to abiotic stress in tea plant (*Camellia sinensis*). *Genomics* 111 1142–1151. 10.1016/j.ygeno.2018.07.009 30031053

[B30] Shi J. Ma C. Qi D. Lv H. Yang T. Peng Q. (2017). Erratum to: transcriptional responses and flavor volatiles biosynthesis in methyl jasmonate-treated tea leaves. *BMC Plant Biol.* 17:136. 10.1186/s12870-017-1076-1075 PMC5549293 28793870

[B31] Shim J. S. Oh N. Chung P. J. Kim Y. S. Choi Y. D. Kim J. K. (2018). Overexpression of OsNAC14 improves drought tolerance in rice. *Front. Plant Sci.* 9:310. 10.3389/fpls.2018.00310 29593766 PMC5855183

[B32] Szabados L. Savouré A. (2010). Proline: a multifunctional amino acid. *Trends Plant Sci.* 15 89–97. 10.1016/j.tplants.2009.11.009 20036181

[B33] Takashi F. K. M. Yasunari F. Taishi U. Riichiro Y. Kazuo S. Kazuko Y. S. (2006). Abscisic acid-dependent multisite phosphorylation regulates the activity of atranscription activator AREB1. *Proc. Natl. Acad. Sci. U S A.* 103 1988–1993. 10.1073/pnas.0505667103 16446457 PMC1413621

[B34] Umezawa T. Nakashima K. Miyakawa T. Kuromori T. Tanokura M. Shinozaki K. (2010). Molecular basis of the core regulatory network in ABA responses: sensing, signaling and transport. *Plant Cell Physiol.* 51 1821–1839. 10.1093/pcp/pcq156 20980270 PMC2978318

[B35] Uno Y. Furihata T. Abe H. Yoshida R. Shinozaki K. Yamaguchi-Shinozaki K. (2000). Arabidopsis basic leucine zipper transcription factors involved in an abscisic acid-dependent signal transduction pathway under drought and high-salinity conditions. *Proc. Natl. Acad. Sci. U S A.* 97 11632–11637. 10.1073/pnas.190309197 11005831 PMC17252

[B36] Wang W. Qiu X. Yang Y. Kim H. S. Jia X. Yu H. (2019). Sweetpotato bZIP transcription factor IbABF4 confers tolerance to multiple abiotic stresses. *Front. Plant Sci.* 10:630. 10.3389/fpls.2019.00630 31156685 PMC6531819

[B37] Wang W. Xin H. Wang M. Ma Q. Wang L. Kaleri N. A. (2016). Transcriptomic analysis reveals the molecular mechanisms of drought-stress-induced decreases in *Camellia sinensis* leaf quality. *Front. Plant Sci.* 7:385. 10.3389/fpls.2016.00385 27066035 PMC4811933

[B38] Wang X. Lu X. Malik W. A. Chen X. Wang J. Wang D. (2020). Differentially expressed bZIP transcription factors confer multi-tolerances in *Gossypium hirsutum* L. *Int. J. Biol. Macromol.* 146 569–578. 10.1016/j.ijbiomac.2020.01.013 31923491

[B39] Wang X. Zhao Q. Ma C. Zhang Z. Cao H. Kong Y. (2013). Global transcriptome profiles of *Camellia sinensis* during cold acclimation. *BMC Genomics* 14:415. 10.1186/1471-2164-14-415 23799877 PMC3701547

[B40] Wang Y. Li T. John S. J. Chen M. Chang J. Yang G. (2018). A CBL-interacting protein kinase TaCIPK27 confers drought tolerance and exogenous ABA sensitivity in transgenic Arabidopsis. *Plant Physiol. Biochem.* 123 103–113. 10.1016/j.plaphy.2017.11.019 29227949

[B41] Wang Z. Cheng K. Wan L. Yan L. Jiang H. Liu S. (2015). Genome-wide analysis of the basic leucine zipper (bZIP) transcription factor gene family in six legume genomes. *BMC Genomics* 16:1053. 10.1186/s12864-015-2258-x 26651343 PMC4676100

[B42] Wang Z. Su G. Li M. Ke Q. Kim S. Y. Li H. (2016). Overexpressing *Arabidopsis* ABF3 increases tolerance to multiple abiotic stresses and reduces leaf size in alfalfa. *Plant Physiol. Biochem.* 109 199–208. 10.1016/j.plaphy.2016.09.020 27721135

[B43] Xia E. H. Li F. D. Tong W. Li P.-H. Wu Q. Zhao H. J. (2019). Tea Plant Information Archive: a comprehensive genomics and bioinformatics platform for tea plant. *Plant. Biotechnol. J.* 17 1938–1953. 10.1111/pbi.13111 30913342 PMC6737018

[B44] Yamaguchi-Shinozaki K. Shinozaki K. (2006). Transcriptional regulatory networks in cellular responses and tolerance to dehydration and cold stresses. *Annu. Rev. Plant Biol.* 57 781–803. 10.1146/annurev.arplant.57.032905.105444 16669782

[B45] Yang W. Lu Z. Xiong Y. Yao J. (2017). Genome-wide identification and co-expression network analysis of the OsNF-Y gene family in rice. *Crop J.* 5 21–31. 10.1016/j.cj.2016.06.014

[B46] Zhang Q. Cai M. Yu X. Wang L. Guo C. Ming R. (2017). Transcriptome dynamics of *Camellia sinensis* in response to continuous salinity and drought stress. *Tree Genet Genomes* 13 78–94. 10.1007/s11295-017-1161-1169

[B47] Zhang S. Chen Y. He X. Du J. Zhang R. Ma Y. (2020). Identification of MYB transcription factors regulating theanine biosynthesis in tea plant using omics-based gene coexpression analysis. *J. Agr. Food Chem.* 68 918–926. 10.1021/acs.jafc.9b06730 31899636

[B48] Zhang X. Wu H. Chen L. Wang N. Wei C. Wan X. (2019). Mesophyll cells’ ability to maintain potassium is correlated with drought tolerance in tea (*Camellia sinensis*). *Plant Physiol. Biochem.* 136 196–203. 10.1016/j.plaphy.2019.01.020 30685699

[B49] Zhou X. Yuan F. Wang M. Guo A. Zhang Y. Xie C. G. (2013). Molecular characterization of an ABA insensitive 5 orthologue in *Brassica oleracea*. *Biochem. Bioph. Res. Co* 430 1140–1146. 10.1016/j.bbrc.2012.12.023 23246838

[B50] Zhou Y. Xu D. Jia L. Huang X. Ma G. Wang S. (2017). Genome-Wide identification and structural analysis of bZIP transcription factor genes in *Brassica napus*. *Genes (Basel)* 8 288–311. 10.3390/genes8100288 29064393 PMC5664138

